# Aminochalcones Attenuate Neuronal Cell Death under Oxidative Damage via Sirtuin 1 Activity

**DOI:** 10.1021/acsomega.3c03047

**Published:** 2023-09-07

**Authors:** Setthawut Apiraksattayakul, Ratchanok Pingaew, Ronnakorn Leechaisit, Veda Prachayasittikul, Waralee Ruankham, Napat Songtawee, Tanawut Tantimongcolwat, Somsak Ruchirawat, Virapong Prachayasittikul, Supaluk Prachayasittikul, Kamonrat Phopin

**Affiliations:** † Center for Research Innovation and Biomedical Informatics, Faculty of Medical Technology, Mahidol University, Bangkok 10700, Thailand; ‡ Department of Chemistry, Faculty of Science, Srinakharinwirot University, Bangkok 10110, Thailand; § Center for Research Innovation and Biomedical Informatics, Faculty of Medical Technology, Mahidol University, Bangkok 10700, Thailand; ∥ Department of Clinical Chemistry, Faculty of Medical Technology, Mahidol University, Bangkok 10700, Thailand; ⊥ Laboratory of Medicinal Chemistry, Chulabhorn Research Institute, and Program in Chemical Science, Chulabhorn Graduate Institute, Bangkok 10210, Thailand; # Center of Excellence on Environmental Health and Toxicology (EHT), Commission on Higher Education, Ministry of Education, Bangkok 10400, Thailand; ¶ Department of Clinical Microbiology and Applied Technology, Faculty of Medical Technology, Mahidol University, Bangkok 10700, Thailand

## Abstract

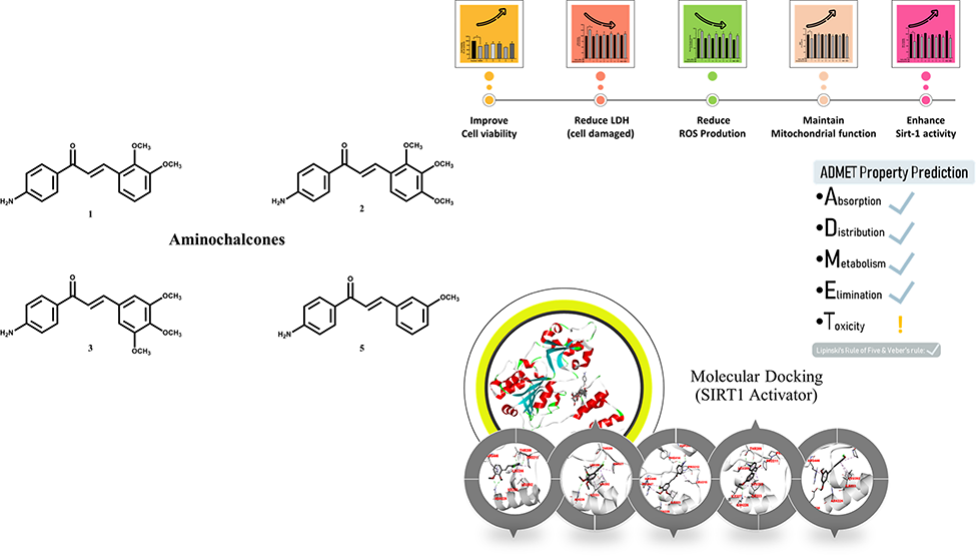

Encouraged by the lack of effective treatments and the dramatic growth in the global prevalence of neurodegenerative diseases along with various pharmacological properties of chalcone pharmacophores, this study focused on the development of aminochalcone-based compounds, organic molecules characterized by a chalcone backbone (consisting of two aromatic rings connected by a three-carbon α,β-unsaturated carbonyl system) with an amino group attached to one of the aromatic rings, as potential neuroprotective agents. Thus, the aminochalcone-based compounds in this study were designed by bearing a –OCH_3_ moiety at different positions on the ring and synthesized by the Claisen–Schmidt condensation. The compounds exhibited strong neuroprotective effects against hydrogen peroxide-induced neuronal death in the human neuroblastoma (SH-SY5Y) cell line (i.e., by improving cell survival, reducing reactive oxygen species production, maintaining mitochondrial function, and preventing cell membrane damage). The aminochalcone-based compounds showed mild toxicity toward a normal embryonic lung cell line (MRC-5) and a human neuroblastoma cell line, and were predicted to have preferable pharmacokinetic profiles with potential for oral administration. Molecular docking simulation indicated that the studied aminochalcones may act as competitive activators of the well-known protective protein, SIRT1, and provided beneficial knowledge regarding the essential key chemical moieties and interacting amino acid residues. Collectively, this work provides a series of four promising candidate agents that could be developed for neuroprotection.

## Introduction

Neurodegenerative diseases have inevitably become a significant part of global public health concerns because of the increasing global life expectancy, aging population,^1,2^ and prevalence of age-related diseases^3^ as well as the incurable nature of these diseases.^4^ Aging drives the loss of neuronal cell integrity (both centrally and peripherally), death of neuronal cells, and structural changes to the brain, which eventually manifest as clinical signs (i.e., cognitive decline and loss of memory) of neurodegenerative diseases.^5–7^ Alzheimer’s disease and Parkinson’s disease are ranked as the top two highest prevalent neurodegenerative conditions among others.^8^

Neurodegeneration is promoted by the accumulative consequences of genetic risk and environmental factors.^9,10^ Overproduction of reactive oxygen species (ROS), such as the superoxide anion (O_2_^•−^), hydrogen peroxide (H_2_O_2_), and hydroxyl radical (HO^•^), induces oxidative stress conditions, which are noted as dominating factors in neurodegeneration. Excessive production of ROS causes prominent harmful events contributing to neuronal cell death, including mitochondrial failure, protein aggregation, and neuroinflammation.^11–15^

Although several clinical drugs are currently available to treat neurodegenerative diseases, most of them were developed for symptomatic treatment as the drugs only alleviate the symptoms or restore the balance of key neurotransmitters without addressing the underlying reasons of disease pathogenesis and progression.^16,17^ Moreover, the clinical side effects and therapeutic ineffectiveness of these drugs still leave gaps in effective therapy.^18–20^ Several compounds with favorable efficacy have been reported for many *in vitro* and *in vivo* neurodegenerative disease models; however, their clinical effectiveness remains questionable, partly because of their unfavorable pharmacokinetics and clinical safety (ADMET—absorption, distribution, metabolism, excretion, and toxicity) profiles. Additionally, the multifactorial nature of the diseases renders their management a complex task since, in many occurrences, these diseases appear sporadically with unclear reasons.^9,21^ These situations collectively make the development of effective drugs for neurodegenerative diseases a challenging issue. To deal with these issues, a novel development strategy of multipotential neuroprotective agents (e.g., antioxidant, antiapoptotic, and anti-inflammatory agents) with disease-modifying nature to delay the disease progression along with preferable ADMET profiles is a key ongoing research area.^22–24^

Recently, sirtuin-1 (SIRT1) has emerged as a potential therapeutic protein target for neurodegenerative diseases, owing to its capacity to enhance neurogenesis and promote cellular longevity. Its neuroprotective effects stem from its ability to modulate pivotal molecular pathways involved in neurodegeneration. Notably, SIRT1 exerts regulatory control over gene expression by modifying the acetylated status of various transcription factors and histone proteins, thereby mitigating the generation and aggregation of misfolded proteins associated with conditions such as amyloid or tau-related diseases. Additionally, SIRT1 exhibits an influence over mitochondrial biogenesis, effectively diminishing oxidative stress, neuroinflammation, and excitotoxicity, which are hallmark features observed in neurodegenerative disorders. Furthermore, SIRT1 plays a crucial role in fostering autophagy, facilitating the clearance of damaged proteins and organelles, while simultaneously governing cellular metabolism to ensure sufficient energy supply for neurons in order to withstand pathological insults. Taken together, targeting SIRT1 holds substantial promise as a comprehensive approach to neuroprotection and represents a potential avenue for combating a range of neurodegenerative disorders.^25^

Naturally occurring compounds have been noted as sources of drugs for decades because of their wide chemical diversity and broad biological properties.^26^ Flavonoids are plant-derived metabolites that exhibit various pharmacological effects. Flavonoid derivatives have been reported as neuroprotectants with many beneficial effects on the brain, including minimizing neuroinflammation, preventing damage from neurotoxins, and enhancing memory, learning, and cognitive performance.^27–29^ Chalcone (1,3-diaryl-2-propen-1-one) is a class of flavonoid derivatives found in many edible plants and spices and is widely used as a scaffold for the synthesis of many pharmacologically active compounds^30,31^ including anti-inflammatory,^32^ antioxidant,^33^ anticancer,^34^ and antibacterial agents.^35^ In addition, several synthesized chalcones have been reported for their neuroprotective effects.^36–38^ Substitution of the amino group on the chalcone core structure has been reported to produce biologically active aminochalcone analogues^39^ with anticancer,^40^ antimicrobial,^39^ anti-inflammatory,^41^ antioxidant,^42^ and neuroprotective properties.^43^ This evidence suggested that the aminochalcone molecule is a potential neuroprotective pharmacophore; however, research supporting its roles against neurodegeneration in human neuronal cell lines remains lacking.

Computational approaches are currently used as facilitating tools for increasing the success rate of drug development.^44^ Molecular docking is commonly used to investigate potential binding modes and interactions of the compounds against their molecular targets.^45,46^*In silico* pharmacokinetic (ADMET) prediction, according to Lipinski’s and Veber’s rules, is also useful for prioritizing the compounds for further successful development.^47^

Herein, a series of five aminochalcone derivatives was synthesized and investigated for their neuroprotective effects against H_2_O_2_-induced oxidative stress in human neuroblastoma SH-SY5Y cells based on morphological changes, cell viability, ROS production, mitochondrial function, cell damage, and SIRT1 activity. Additionally, molecular docking was performed to reveal the possible binding modes and interactions of the compounds with the SIRT1 target. *In silico* ADMET prediction was performed to demonstrate drug-likeness and potential for further development as neuroprotective agents.

## Results

### Synthesis of Aminochalcones

**Figure 1:**
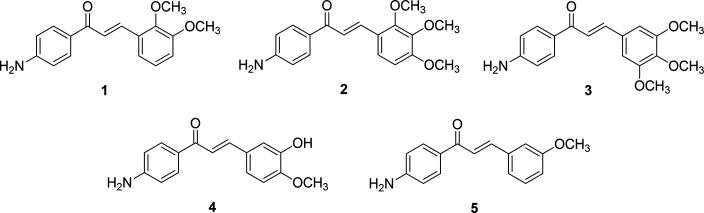
Chemical structures of synthesized aminochalcones **1**–**5**.

A series of *para*-aminochalcones (**1**–**5**) was synthesized by the Claisen–Schmidt condensation of 4-aminoacetophenone **A** and the corresponding benzaldehyde **B** with NaOH as the base (Scheme S1).^23,48,49^ All compounds were characterized by using ^1^H nuclear magnetic resonance (NMR), ^13^C NMR, Fourier transform infrared (FTIR), and high-resolution mass spectroscopy (HRMS). Chemical structures of the synthesized aminochalcones (**1**–**5**) are shown in Figure 1.

### Synthesized Aminochalcones Exhibited Low Cytotoxicity against the Normal Embryonic Lung (MRC-5) Cell Line

The cytotoxicity of the synthesized compounds (**1**–**5**) was investigated against the normal embryonic lung (MRC-5) cell line (Table 1). All compounds showed low cytotoxicity toward the tested normal cells (IC_50_ = 70.34–116.34 μM) when compared with that of the reference drug, doxorubicin (IC_50_ = 3.03 μM).

**Table 1:** Cytotoxic Activity (IC_50_, μM) of Aminochalcones (**1**–**5**) against the Normal Embryonic Lung (MRC-5) Cell Line

Compound	Cytotoxic activity (μM)
**1**	79.27 ± 6.63
**2**	99.31 ± 4.15
**3**	70.34 ± 3.78
**4**	115.30 ± 1.05
**5**	116.34 ± 8.08
doxorubicin^a^	3.03 ± 0.09

a Doxorubicin was used as the reference drug.

### Aminochalcones Improved Cell Viability in H_2_O_2_-Induced SH-SY5Y Cells

**Figure 2:**
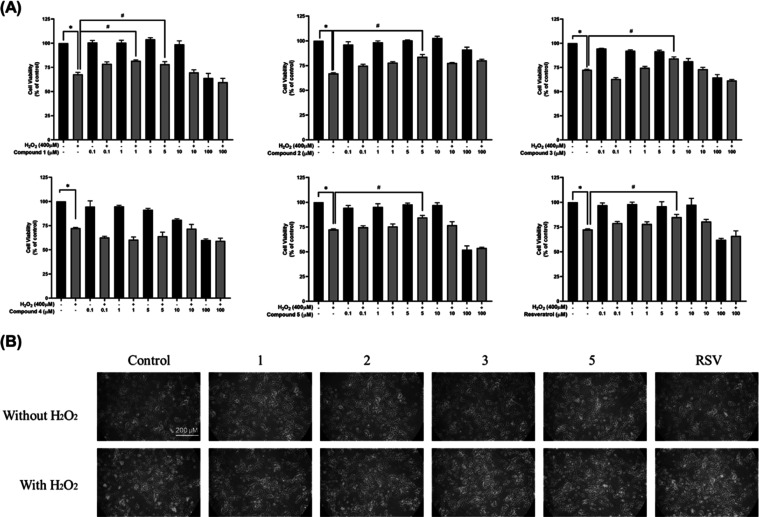
Effect of aminochalcones (**1**–**5**) on SH-SY5Y cell viability and morphology. (A) Cell viability was evaluated by using the MTT assay at 570 nm. The viability of the cells was expressed as the percentage relative to the viability of the control group. Statistical analysis was performed utilizing one-way ANOVA; data are reported as mean ± SEM *P < 0.05 compared with the control; #*P* < 0.05 compared with the H_2_O_2_. (B) Effect of selected aminochalcones on the cell morphology. Morphological transformation of the SH-SY5Y cells was seen using an inverted light microscope (Olympus Corporation, Tokyo, Japan) at ×20 magnification. Scale bar = 200 μm.

The effects of aminochalcones (**1**–**5**) on the viability of neuroblastoma SH-SY5Y cells were assessed using the MTT (MTT, 3-(4,5-dimethylthiazol-2-yl)-2,5-diphenyltetrazolium bromide) assay. Cells in the absence of H_2_O_2_ were used as the control group. Treating cells with compounds alone at concentrations from 0.1 to 10 μM showed no effects on cell viability except for compounds **3** and **4**, where concentrations at 10 μM became hazardous to the treated cells (Figure 2A). All compounds at a high concentration (100 μM) notably reduced the cell viability when compared with that of the control. Furthermore, the exposure to 400 μM H_2_O_2_ triggered neuronal cell death by 27.49–32.96%.

To represent the oxidative stress model, the cells were pretreated and incubated with various concentrations (0.1–100 μM) of each compound for 3 h, followed by exposure to 400 μM H_2_O_2_ for an additional period of 24 h. Cell viability was then evaluated to assess the neuroprotective potential of each compound. When compared with the effect of 400 μM H_2_O_2_ alone, the pretreatment with aminochalcone derivatives (**1**–**3** and **5**) at 5 μM effectively restored the cell viability up to 78.25–84.66% (Figure 2A). A similar finding was observed for pretreatment with resveratrol (84.99%), which is a reference antioxidant compound that is known to protect cell viability. Accordingly, these four compounds (**1**–**3** and **5**) at a concentration of 5 μM were chosen for additional experimental studies to uncover their potential neuroprotective pathways.

### Selected Aminochalcones Prevented Morphological Alterations in H_2_O_2_-Induced SH-SY5Y Cells

Bright-field microscopy was employed to observe the cell morphology to determine the effect of four chosen compounds (**1**–**3** and **5**) on SH-SY5Y cells. Abnormal morphological changes were clearly apparent in cells exposed to 400 μM H_2_O_2_ compared with the control. The changes were shown as cellular shrinkage and unattachment, as well as subsequent transformation into a tiny-rounded shape (Figure 2B). By contrast, when the cells were pretreated with compounds (**1**–**3** and **5**) or resveratrol at 5 μM concentration for 3 h before exposure to H_2_O_2_, the morphological alterations were reduced, as shown by less cellular damage, a more adherent appearance, and appropriate cell growth when compared with those were not pretreated (Figure 2B).

### Reduction in H_2_O_2_-Induced ROS Production in SH-SY5Y Cells by Pretreatment with Selected Aminochalcones

**Figure 3:**
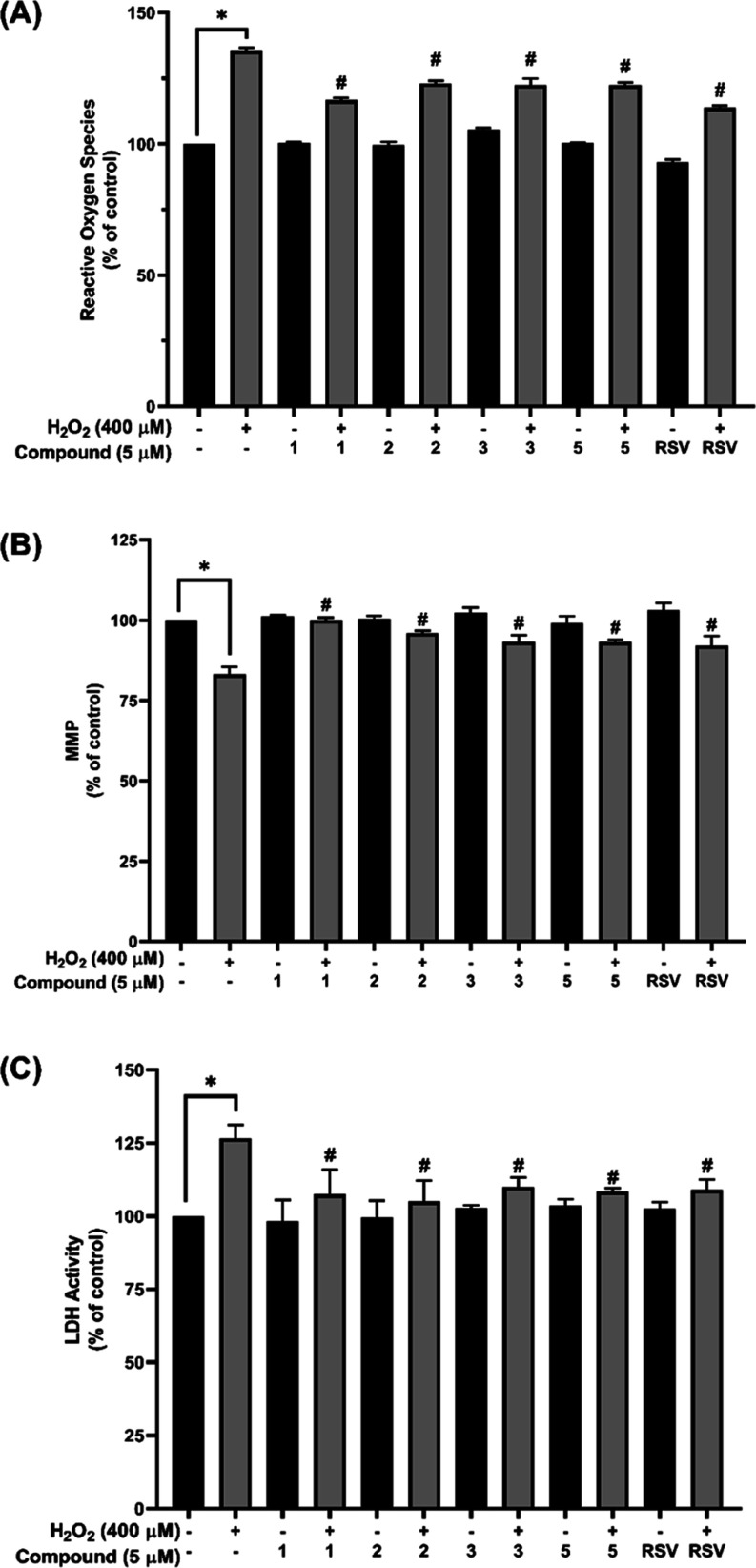
Protective effects of four selected aminochalcones against the H_2_O_2_-induced pathological changes in SH-SY5Y cells: (A) Intracellular ROS production, (B) MMP, and (C) LDH activity. SH-SY5Y cells were pretreated with 5 μM aminochalcones or resveratrol before exposure to 400 μM H_2_O_2_ for an additional 24 h before the measurements. Results were expressed as percentages compared with the control group. Statistical analysis was performed utilizing one-way ANOVA; data are reported as mean ± SEM; *P < 0.05 compared with the control; #*P* < 0.05 compared with the H_2_O_2_ treatment.

Intracellular ROS levels were measured using a fluorescence probe, 2′-7′-dichlorodihydrofluorescein diacetate (DCFDA), to prove that the selected aminochalcones (**1**–**3** and **5**) can reduce ROS production and oxidative stress caused by H_2_O_2_. After exposure to 400 μM H_2_O_2_ for 24 h, the intensity of the fluorescence significantly increased to 135.54% ± 1.9 when compared with that of the control (Figure 3A). Conversely, the fluorescence intensity significantly decreased for cells pretreated with the selected compounds or resveratrol before exposure to H_2_O_2_ (116.71 ± 1.2 or 122.97 ± 2.0%, respectively; Figure 3A) when compared with that of the control group.

### Selected Aminochalcones Helped Maintain Mitochondrial Membrane Potential in H_2_O_2_-Induced SH-SY5Y Cells

A decline in mitochondrial membrane potential (MMP) has been linked to mitochondrial malfunction and apoptosis.^50^ The aminochalcones (**1**–**3** and **5**) were investigated for their protective roles in mitochondrial dysfunction in by measuring the fluorescence signal from Rhodamine-123 dye used to label the active membrane of the SH-SY5Y cells. Exposure to 400 μM H_2_O_2_ caused a decline in MMP to 83.03 ± 5.6% when compared with that of the control group (Figure 3B). However, the MMP was significantly increased from (93.17 ± 1.2 to 99.95 ± 1.3%) in cells pretreated with 5 μM of the investigated compounds or resveratrol before exposure to H_2_O_2_ (Figure 3B).

### Decrease of Neuronal Damage and Lactate Dehydrogenase Activity in H_2_O_2_-Induced SH-SY5Y Cells by Pretreatment with Selected Aminochalcones

The effects of selected compounds (**1**–**3** and **5**) on lactate dehydrogenase (LDH) activity in the growth media were assessed. LDH serves as a signal for cellular damage. In the presence of cell membrane leakage or rupture, LDH leaks into the culture media, causing increased activity.^51^ Exposure to 400 μM H_2_O_2_ caused an elevation of LDH activity (126.64 ± 7.7%), whereas a reduction of the LDH activity was observed for cells pretreated with the studied compounds or resveratrol for 3 h before H_2_O_2_ exposure (105.07 ± 14.2 to 109.99 ± 5.6%) when compared with that of the control group (Figure 3C).

### Selected Aminochalcones Enhanced SIRT1 Activity in H_2_O_2_-Induced SH-SY5Y Cells

**Figure 4:**
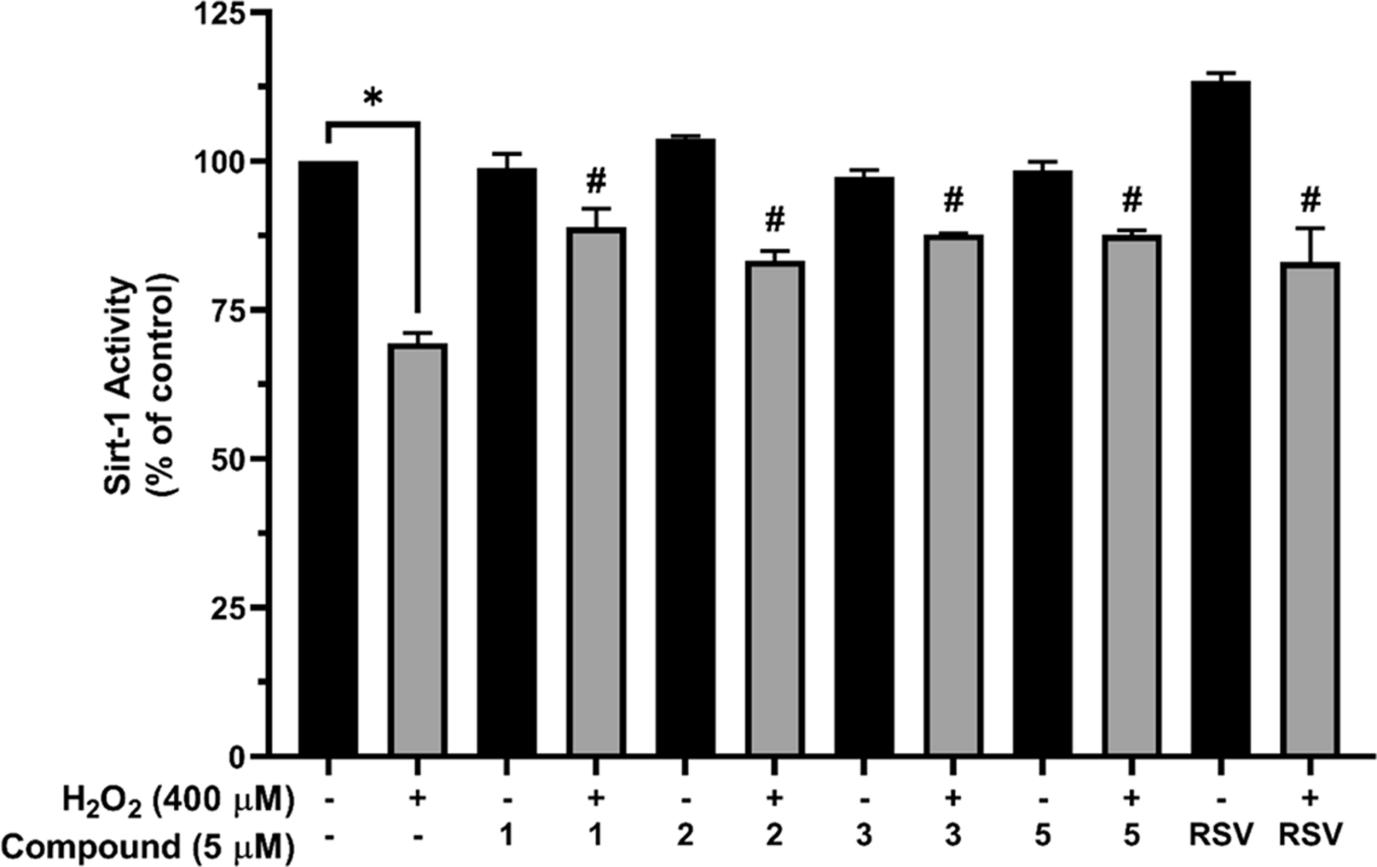
Effect of four selected aminochalcones on SIRT1 activity in SH-SY5Y cells. SH-SY5Y cells were extracted in the lysate buffer, followed by determination of the amount of protein using the Bradford protein assay. Proteins were then analyzed for SIRT1 activity using the SIRT1 assay kit. SIRT1 activity of the cells was expressed as a percentage relative to the control. Statistical analysis was performed utilizing one-way ANOVA; data are reported as mean ± SEM; *P < 0.05 compared with the control; #*P* < 0.05 compared with the H_2_O_2_ treatment.

SIRT1 activity in SH-SY5Y cells was examined. Decreased SIRT1 activity was observed (69.44 ± 3.40%) in the H_2_O_2_-treated cells after 24 h of incubation when compared with that of the control (Figure 4). By contrast, pretreatment with chosen compounds (**1**–**3** and **5**) for 3 h before H_2_O_2_ exposure maintained SIRT1 activity within 83.30 ± 2.8 to 88.97 ± 5.2% when compared with that of the control group (Figure 4).

### Molecular Docking Study Revealed Possible Binding Modalities between Selected Aminochalcones against the SIRT1 Activator-Binding Site

**Figure 5:**
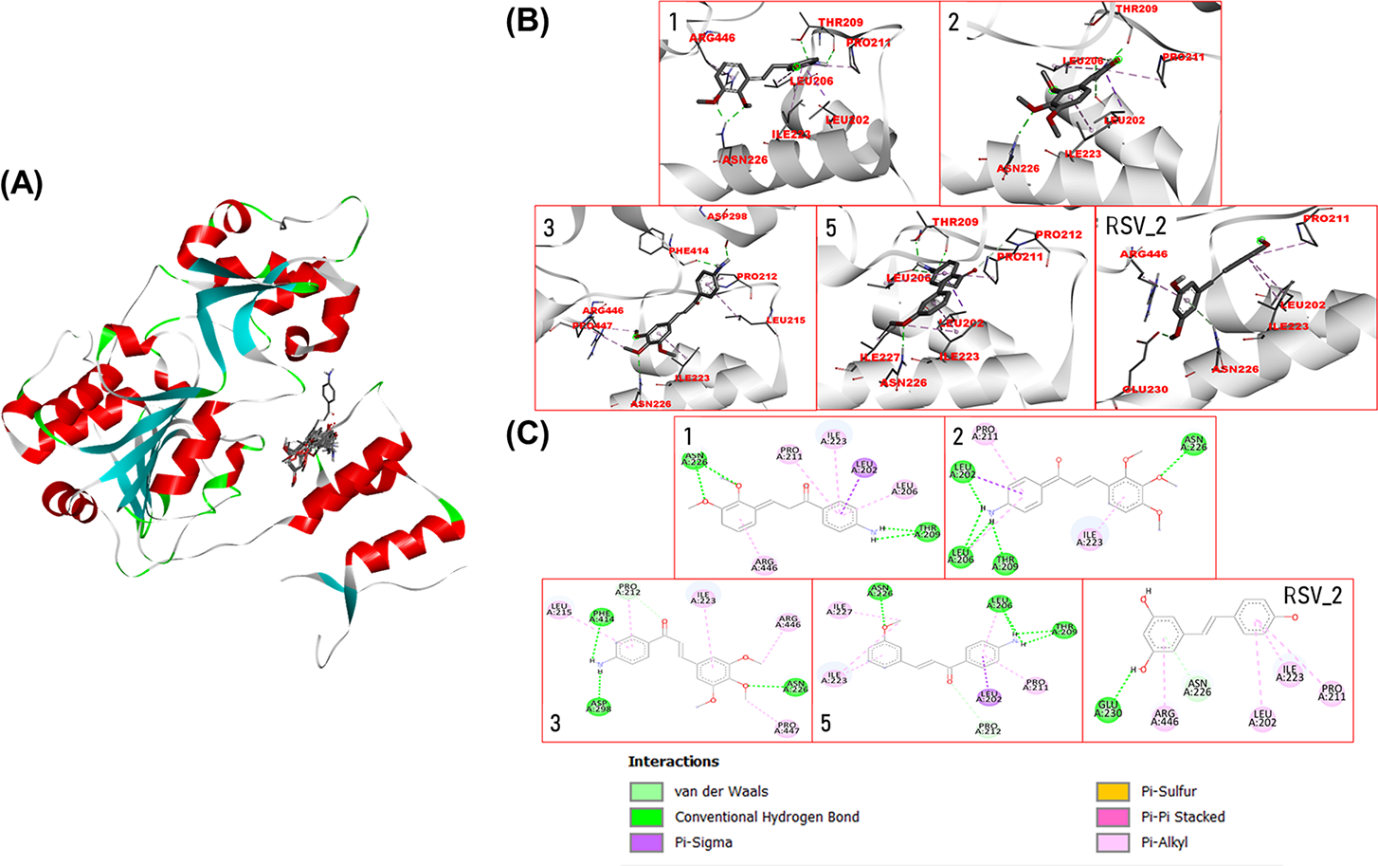
Possible binding modalities and binding interactions between selected aminochalcones against the SIRT1 activator-binding site. (A) Binding pockets of four aminochalcones and cocrystallized ligand (resveratrol) within the SIRT1 protein (PDB: 5BTR). (B) Possible binding modalities of each aminochalcone (**1**, **2**, **3**, and **5**) or resveratrol at the activator-binding site of SIRT1 protein. (C) Two-dimensional protein–ligand interaction diagrams of aminochalcones (**1**, **2**, **3**, and **5**) and resveratrol within the SIRT1 activator-binding site. The participating residues are shown as colored circles according to the type of interaction.

The potential binding modes of selected aminochalcones (**1**–**3** and **5**) against the target protein, SIRT1 (Protein Data Bank [PDB] code 5BTR),^52^ were simulated using molecular docking. First, redocking was performed using three cocrystallized resveratrol molecules to confirm the validity of the docking process. The calculated mean binding free energies for resveratrol-1 (RSV1), resveratrol-2 (RSV2), and resveratrol-3 (RSV3) from the redocking were −7.46, −7.55, and −7.41 kcal/mol, respectively. A root-mean-square deviation value of 2.0 was obtained as a redocking result, indicating the reliability of the docking protocol for further simulation using the compounds of interest. Multiple docking runs of the investigated compound were undertaken; however, only the model with the highest ranking in each compound cluster was chosen. Docking poses revealed that all aminochalcones could occupy the same activator-binding region of the SIRT1 protein in a similar manner to that of the resveratrol molecules (RSV1 and RSV2), Figure 5A. Moreover, the binding free energy values of the docked compounds **1**, **2**, **3**, and **5** (−9.11, −9.34, −9.07, and −8.53 kcal/mol, respectively) were lower than those of the resveratrol cluster molecules (RSV1 and RSV2).

Docking poses (Figure 5B) and two-dimensional ligand–protein interaction diagrams (Figure 5C) were created to clarify key interactions contributing to the binding modes of the compounds toward target SIRT1. Here, RSV2 was selected as a reference model because this had the lowest binding free energy among the three resveratrol molecules. All the aminochalcone compounds shared several common interacting amino acid residues with RSV2 (i.e., LEU202, PRO211, ILE223, and ASN226). Resveratrol binding was demonstrated to be primarily mediated *viapi*-alkyl interactions (i.e., LEU202, PRO211, ILE223, and ARG446) as well as by van der Waals forces (i.e., ASN226) and hydrogen bonding (i.e., GLU230). Likewise, the binding mechanisms of the investigated compounds were mostly mediated *viapi*-alkyl, *pi*-sigma, and van der Waals interactions. This finding suggested that the aminochalcones and the well-known SIRT1 activator resveratrol share a similar binding manner.

### Selected Aminochalcones Possessed Favorable Predictions for Pharmacokinetic Properties

Three web-based *in silico* tools were used to predict the properties of the studied compounds: SwissADME for physicochemical properties and drug-likeness, pkCSM for pharmacokinetic properties, and ProTox-II for toxicity. Prediction results are provided in Table 2. The results showed that the selected compounds (**1**, **2**, **3**, and **5**) are drug-like compounds as judged by the parameters of Lipinski’s and Veber’s rules. All compounds were predicted to be poorly water-soluble with optimal lipophilicity for oral administration (log *P* value < 5, water solubility of 0.055–0.121, and intestinal absorption percentage of 92.22–95.27%).^53^ Additionally, these compounds showed a moderate volume of potential distribution (human Vd of 0.089–0.384 log L/kg) and a moderate potential to reach the target site of action in the central nervous system (CNS), as shown by their blood–brain barrier (BBB) and CNS permeability. However, none of the compounds were substrates of the main metabolizing enzymes CYP2D6 and CYP3A4, which belong to a subclass of the cytochrome P450 family responsible for the metabolism of >90% of drugs.^54^ Additionally, these compounds were not inhibitors of CYP1A2, CYP2C19, or CYP3A4, except for compound **5**, which was not a CYP3A4 inhibitor. These metabolic parameters suggested an alternative metabolic fate and potential drug–drug interactions with other drugs/substances.^53^ For drug elimination, the total clearance of the compounds was 0.223–0.606 log mL/min/kg, and none of the compounds were substrates of the renal organic cation transporter 2 (OCT2).

**Table 2:** Prediction of Physicochemical Characteristics, Pharmacokinetic Properties, and Toxicity of Selected Aminochalcones (i.e., **1**, **2**, **3**, and **5**)^a^

Compound	**1**	**2**	**3**	**5**
**Physicochemical Properties**				
molecular weight	283.327	313.353	313.353	253.301
ilog P	3.1821	3.1907	3.1907	3.1735
rotatable bonds	5	6	6	4
H-bond acceptors	4	5	5	3
H-bond donors	1	1	1	1
Lipinski’s rule	pass	pass	pass	pass
Veber’s rule	pass	pass	pass	pass
**Absorption**				
water solubility (log mol/L)	0.082	0.077	0.077	0.055
intestinal absorption (% absorbed)	95.279	94.811	94.276	94.777
**Distribution**				
VDss^b^ (human) (log L/kg)	0.292	0.111	0.089	0.384
BBB^c^ permeability (log BB)	–0.139	–0.312	–0.290	–0.102
CNS^d^ permeability (log PS)	–2.202	–2.351	–2.336	–2.03
**Metabolism**				
CYP2D6 substrate	no	no	no	no
CYP3A4 substrate	no	no	no	no
CYP1A2 inhibitor	yes	yes	yes	yes
CYP2C19 inhibitor	yes	yes	yes	yes
CYP3A4 inhibitor	yes	yes	yes	no
**Excretion**				
total clearance (log mL/min/kg)	0.331	0.606	0.239	0.223
renal OCT2 substrate	no	no	no	no
**Toxicity**				
LD_50_^e^ (mg/kg)	1000 moderately toxic	2100 slightly toxic	3000 slightly toxic	3000 slightly toxic

a The prediction was performed using SwissADME (http://www.swissadme.ch/), pkCSM (http://biosig.unimelb.edu.au/pkcsm/), and ProTox-II (http://tox-new.charite.de/protox_II/).

b VDss, volume of distribution.

c BBB, blood–brain-barrier.

d CNS, central nervous system.

e LD_50_, 50% lethal dose.

## Discussion

The continual global increase in the aging population has supported the expanding prevalence of neurodegenerative disorders.^55^ Accordingly, the development of neuroprotective agents that can prevent or delay the devastating effects and disease progression is urgently needed.^3,56^ The current development of multifunctional neuroprotective compounds for addressing challenging issues in this area has become more popular, and antioxidant compounds with multiple neuroprotective actions have gained considerable attention. In this study, a series of five aminochalcone derivatives (**1**–**5**) was synthesized and investigated for their cytotoxicities to a normal embryonic lung (MRC-5) cell line as well as evaluate the neuroprotective capability in the SH-SY5Y cell line against H_2_O_2_-induced oxidative stress. Computational simulation and prediction were also performed to elucidate the possible binding behaviors and pharmacokinetic profiles.

H_2_O_2_ is frequently used to generate cellular oxidative stress and induce intracellular biological changes. It is considered a highly potent neurotoxic compound that is used as an inducer in many neurodegenerative disease-induced models^57,58^ because of its rapid generation of exogenous free radicals that strongly acts as ROS.^59,60^ Treating SH-SY5Y cells with H_2_O_2_ at concentrations of 200–500 μM for 24 h has been shown to produce a dose-dependent loss of cell viability, and treatment with 400 μM can induce approximately 3–35% cell death.^61^ Accordingly, a concentration of 400 μM H_2_O_2_ was used to induce intracellular oxidative stress conditions in this study.

Our results showed that exposure to 400 μM H_2_O_2_ could induce cell death and morphological changes and elevate intracellular ROS levels, mitochondrial dysfunction, and cell membrane damage as well as reduce SIRT1 activity in the exposed SH-SY5Y cells, whereas the protective effects against these devastating events can be observed for the cells pretreated with the studied aminochalcones. The effect of the studied compounds (**1**–**5**) on the cell viability of SH-SY5Y cells was investigated. Treatment with the tested compounds (**1**–**5**) alone at concentrations <5 μM did not affect the cell viability, although cell death was observed when the cells were treated with higher concentrations (10–100 μM), Figure 2A.

The neuroprotective effects of compounds **1**–**5** were studied by pretreating the cells with the compounds at various concentrations (0.1–100 μM for 3 h) before the oxidative stress induction (exposure to 400 μM H_2_O_2_ for an additional 24 h). The findings showed that four compounds (**1**, **2**, **3**, and **5**) at 5 μM significantly improved the cell viability when compared with the nonpretreated control group, Figure 2A. However, compound **4** displayed low protective potential at 5 μM, Figure 2A. This outcome may be attributed to the presence of the –OH functional group in compound **4**, which sets it apart from other compounds. The presence of the –OH group may impede the compound’s interactions with the target receptor and hinder the cellular uptake, resulting in reduced affinity for neuroprotective pathways and restricted access to intended cellular targets. Furthermore, the –OH group may induce chemical modifications that alter the compound’s bioavailability, potentially compromising its ability to exert desired neuroprotective effects. Further research is warranted to validate these hypotheses and to acquire a more comprehensive understanding of the underlying mechanisms involved. Accordingly, the four aminochalcones (**1**, **2**, **3**, and **5** at 5 μM) with promising neuroprotective effects were selected for subsequent experiments.

Aminochalcones **1**, **2**, **3**, and **5** were further investigated for their effects on cell morphology, intracellular ROS production, mitochondrial function, and cell membrane damage. Interestingly, all tested compounds showed impressive protective effects with pretreatment at 5 μM before H_2_O_2_ exposure. Pretreatment with these compounds prevented morphological aberration (Figure 2B), reduced intracellular ROS production, reduced cell membrane damage, and prevented mitochondrial dysfunction (Figure 3A–C) when compared with the cells treated with H_2_O_2_ alone. This suggested that compounds **1**, **2**, **3**, and **5** could protect the neuronal cells from the H_2_O_2_-induced oxidative stress, which is the main factors related to pathogenesis and progression of neuronal cell death (i.e., oxidative stress, mitochondrial dysfunction, and cellular damage) in various neurodegenerative diseases, including Alzheimer’s and Parkinson’s diseases.^62,63^ Therefore, aminochalcone-based compounds that we studied have the potential to provide neuroprotection. However, our present study primarily centered on evaluating these compounds within the context of H_2_O_2_-induced oxidative damage, and it is imperative to investigate their effectiveness in mitigating neurodegeneration resulting from the accumulation of degenerative proteins in neurons. This aspect warrants careful examination to establish the therapeutic potential of aminochalcone-based compounds in addressing neurodegenerative processes associated with protein aggregation.

The effect of aminochalcones **1**, **2**, **3**, and **5** on SIRT1 activity was then investigated to elucidate the possible mechanisms underlying the neuroprotective effects of these compounds. SIRT1, also known as NAD-dependent deacetylase SIRT1, is a sirtuin family protein encoded by the *SIRT1* gene. SIRT1 is expressed in many organs, including the brain, and acts as a transcription factor for several protective pathways. Downregulation of *SIRT1* expression and decreased SIRT1 levels are associated with the pathogenesis of neurodegenerative diseases since these events might enhance oxidative stress and inflammation, whereas the overexpression of SIRT1 supports protective effects (i.e., enhanced cell viability, decreased cell death, decreased proinflammatory cytokine levels, increased antioxidant mechanisms, and regulated mitochondrial functions). Accordingly, SIRT1 activators have emerged as one of the most promising disease-modifying agents for the management of neurodegenerative diseases.^64–66^ One of the key mechanisms by which SIRT1 exerts its neuroprotective effects is through the modulation of important molecular pathways involved in neurodegeneration. SIRT1 possesses deacetylase activity and can modify the acetylation status of various proteins, including transcription factors and histones, thereby regulating gene expression. By these pathways, SIRT1 can reduce the generation and aggregation of misfolded proteins that are essential to the etiology of neurodegenerative diseases, including amyloid beta and tau. SIRT1 activation has also been demonstrated to reduce oxidative stress, neuroinflammation, and excitotoxicity, the frequent hallmarks of many neurodegenerative diseases, by upregulating antioxidant proteins like FOXO3a, SOD2, and CAT. Moreover, SIRT1 plays a vital role in maintaining cellular health and prolonging lifespan. It promotes the activation of autophagy, a cellular process responsible for clearing damaged proteins and organelles, through AMPK, HIF-1α, and PGC1α activation. By enhancing autophagic activity, SIRT1 facilitates the removal of toxic protein aggregates and damaged mitochondria, thus reducing neuronal stress and improving cellular viability. Additionally, SIRT1’s involvement in cellular metabolism and energy homeostasis contributes to neuroprotection. SIRT1 activation was also reported to be involved in cell membrane repair process.^25,67–69^ In this study, our results indicated that SIRT1 activity decreased on the exposure to 400 μM H_2_O_2_. Conversely, pretreatment with aminochalcones significantly restored the SIRT1 activity (Figure 4). This suggests one of the promising underlying protective mechanisms of our compounds. However, in this study, our focus was on the activity of the SIRT1 protein; therefore, further investigation is still required to understand the exact mechanism of our compounds. Furthermore, molecular docking revealed the binding modalities and possible ligand–protein interactions between the studied compounds (**1**, **2**, **3**, and **5**) and the SIRT1 target (PDB code: 5BTR)^52,70^ (Figure 5). The simulations suggested that all of the studied compounds could occupy the same binding site as that of resveratrol, a well-known SIRT1 activator, and may act as competitive SIRT1 activators. This was corroborated by the sharing of certain critical *pi*-alkyl interactions formed between key amino acid residues (i.e., LEU202, PRO211, ILE223, and ASN226) of the enzyme and the benzene ring of the aminochalcones. Notably, all compounds displayed a binding free energy lower than those of the resveratrol molecules, which indicated that they might have a superior SIRT1 binding effect. Furthermore, the hydrogen bonding between the oxygen atoms of methoxyl (–OCH_3_) groups on the benzene ring and ASN226 residue seems to be a unique feature for effective binding of these compounds, unlike with resveratrol where the ligand binds to the ASN226 residue through van der Waals forces (Figure 5C). This finding was supported by studies, suggesting that the site-directed modification of ASN226 leads to a decreased rate of SIRT1 deacetylation in response to resveratrol stimulation, which supported the necessity of the ASN226 interaction in SIRT1 activation. Furthermore, the amino group (–NH_2_) on the benzene ring of the chalcone core may be essential for these binding characteristics, as demonstrated by the presence of additional hydrogen bonds formed between H atoms of the amino moiety and of THR209 and LEU206 (for compounds **1**, **2**, and **5**) and of PHE414 and ASP298 (for compound **3**), whereas these binding interactions are absent for resveratrol (Figure 5C). Additionally, the most potent compound (**1**) is the only one that formed an additional *pi*-alkyl interaction between the second benzene ring and the ARG446 residue, which might contribute to its higher potency. Collectively, the neuroprotective impact of selected aminochalcones (**1**, **2**, **3**, and **5**) may be partly due to their SIRT1-activating properties.

Finally, most innovative drug developments have been recognized to fail owing to unsatisfactory pharmacokinetics, bioavailability, and clinical safety in the late stages of the development pipeline.^71–73^ Delivery of drugs to certain target organs, such as the CNS, where the BBB is a key barrier, is considered a problematic issue in neuroprotective pharmaceuticals.^74^ Results from *in silico* predictions suggested that all aminochalcones (**1**, **2**, **3**, and **5**) are drug-like compounds with moderate CNS penetration and BBB-crossing abilities, which supported their potential for further development as neuroprotective agents. The predictions also suggested that most of the compounds could be possibly administered as oral drugs because of their suitable lipophilicity (ilog *P* < 5) and intestinal absorption ability (92.22–95.27%). All compounds had low predicted toxicity and were considered safe compounds, except for compound **1**, which had moderate toxicity. These prediction results are supported by our cytotoxicity MTT assay showed that these compounds were mildly cytotoxic but only caused harmful effects on cell viability at high doses. However, these compounds were predicted as inhibitors of many metabolizing enzymes (i.e., CYP1A2, CYP2C19, and CYP3A4); therefore, this should be concerned if coadministered with other drugs to avoid drug–drug interactions and undesired side effects.^53^ Collectively, these predictions suggested that the selected aminochalcones (**1**, **2**, **3**, and **5**) are potentially suitable for oral drug development, with some cautions regarding their toxicities.

## Conclusions

Based on the findings of our study, it has been determined that four specific compounds (**1**, **2**, **3**, and **5**) exhibit neuroprotective effects against H_2_O_2_-induced oxidative stress in human neuroblastoma SH-SY5Y cells through promotion of cell viability, mitochondrial function, antioxidant activity, and SIRT1 activity. Henceforth, these compounds exhibit considerable potential for further investigation into their neuroprotective effects, as well as for conducting in-depth studies on the relationship between their structural attributes and biological activities. Such endeavors hold promise for their prospective application in medicinal contexts in the future. Somehow, our current study focused on the assessment of these compounds in the context of H_2_O_2_-induced oxidative damage, and it is necessary to expand the investigation to effectiveness against neurodegeneration caused by accumulation of degenerative proteins in neurons. Moreover, our primary focus was on the SIRT1 protein, which serves as an upstream regulator of protective mechanisms. It is important to note that the expression and activity of other protective proteins associated with the SIRT1 protein were not examined in this study. Therefore, further attention should be directed toward the behavior of these protective proteins in order to gain a more comprehensive understanding of the effects of compounds on signaling cascades within neuronal cells. Additionally, *in silico* tools were employed to predict the pharmacokinetic properties, drug-likeness, and toxicity of the compounds. While these results were based on theoretical predictions, it is imperative that further experimental studies could be conducted using *in vivo* models and clinical trials in order to more accurately comprehend their therapeutic performances.

## Materials and Methods

### General Chemical Purification and Analysis

Column chromatography was performed using silica gel 60 (70–230 mesh ASTM). Analytical thin-layer chromatography (TLC) was performed on silica gel 60 F_254_ aluminum sheets. Melting points were determined by using a Griffin melting point apparatus and were uncorrected. ^1^H and ^13^C NMR spectra were recorded on a Bruker AVANCE NEO 500 NMR spectrometer. FTIR spectra were obtained using a universal attenuated total reflectance apparatus attached to a PerkinElmer Spectrum One spectrometer. HRMS spectra were recorded on a Bruker Daltonics (microTOF).

#### Synthesis of Aminochalcones **1**–**5**

A mixture of aminoacetophenone **A** (6 mmol) and benzaldehyde derivatives **B** (6 mmol) in ethanol (15 mL) was stirred at 4 °C and then 40% NaOH (5 mL) was added dropwise. The mixture was stirred at room temperature for 4–24 h and monitored using TLC. The reaction mixture was neutralized with 2 M HCl, then the precipitate was filtered, and washed with cold water and cold ethanol to give compounds **1**–**5**.

#### (*E*)-1-(4-Aminophenyl)-3-(2,3-dimethoxyphenyl)prop-2-en-1-one (Compound **1**)^47,73^

Synthesized from 4-aminoacetophenone and 2,3-dimethoxybenzaldehyde. Yellow solid, 93% yield; mp 133–134 °C; IR (neat) cm^–1^: 3461, 3351, 3229, 1626, 1579. ^1^H NMR (500 MHz, CDCl_3_): δ 3.88, 3.89 (s, 6H, 2 × OC*H*_3_), 4.14 (br s, 2H, N*H*_2_), 6.70 (d, *J* = 8.5 Hz, 2H, Ar*H*), 6.95 (dd, *J* = 8.1 Hz, 1H, Ar*H*), 7.08 (t, *J* = 8.1 Hz, 1H, Ar*H*), 7.27 (dd, *J* = 8.6, 1.0 Hz, 1H, Ar*H*), 7.60 (d, *J* = 15.8 Hz, 1H, COC*H*=CHAr), 7.93 (d, *J* = 8.6 Hz, 2H, Ar*H*), 8.05 (d, *J* = 15.8 Hz, 1H, COCH=C*H*Ar). ^13^C NMR (125 MHz, CDCl_3_): δ 56.1, 61.4, 113.9, 114.1, 119.8, 123.9, 124.3, 128.9, 129.8, 131.3, 138.1, 148.9, 151.1, 153.4, 188.7 HRMS-TOF: [M + Na]^+^ 306.1102 (calcd for C_17_H_17_NNaO_3_: 306.1101).

#### (*E*)-1-(4-Aminophenyl)-3-(2,3,4-trimethoxyphenyl)prop-2-en-1-one (Compound **2**)^47^

Synthesized from 4-aminoacetophenone and 2,3,4-trimethoxybenzaldehyde. Yellow solid, 92% yield; mp 125–126 °C; IR (neat) cm^–1^: 3458, 3347, 3231, 1627, 1579. ^1^H NMR (500 MHz, CDCl_3_): δ 3.89, 3.90, 3.93 (s, 9H, 3 × OC*H*_3_), 4.15 (br s, 2H, N*H*_2_), 6.69 (d, *J* = 8.6 Hz, 2H, Ar*H*), 6.71 (d, *J* = 8.7 Hz, 1H, Ar*H*), 7.37 (d, *J* = 8.8 Hz, 1H, Ar*H*), 7.56 (d, *J* = 15.8 Hz, 1H, COC*H*=CHAr), 7.92 (d, *J* = 8.8 Hz, 2H, Ar*H*), 7.95 (d, *J* = 16.0 Hz, 1H, COCH=C*H*Ar). ^13^C NMR (125 MHz, CDCl_3_): δ 56.2, 61.1, 61.5, 107.7, 114.1, 121.6, 122.6, 123.9, 129.1, 131.1, 138.6, 142.7, 151.0, 153.8, 155.5, 188.7 HRMS-TOF: [M + Na]^+^ 336.1205 (calcd for C_18_H_19_NNaO_4_: 336.1206).

#### (*E*)-1-(4-Aminophenyl)-3-(3,4,5-trimethoxyphenyl)prop-2-en-1-one (Compound **3**)^47^

Synthesized from 4-aminoacetophenone and 3,4,5-trimethoxybenzaldehyde. Yellow solid, 87% yield; mp 152–153 °C; IR (neat) cm^–1^: 3459, 3359, 3232, 1626, 1583. ^1^H NMR (500 MHz, CDCl_3_): δ 3.89, 3.92 (s, 9H, 3 × OC*H*_3_), 4.18 (br s, 2H, N*H*_2_), 6.70 (d, *J* = 8.6 Hz, 2H, Ar*H*), 6.85 (s, 2H, Ar*H*), 7.41 (d, *J* = 15.5 Hz, 1H, COC*H*=CHAr), 7.69 (d, *J* = 15.6 Hz, 1H, COCH=C*H*Ar), 7.93 (d, *J* = 8.6 Hz, 2H, Ar*H*). ^13^C NMR (125 MHz, CDCl_3_): δ 56.4, 61.1, 105.6, 114.1, 121.5, 128.7, 131.0, 131.2, 140.2, 143.5, 151.2, 153.6, 188.2. HRMS-TOF: [M + Na]^+^ 336.1203 (calcd for C_18_H_19_NNaO_4_: 336.1206).

#### (*E*)-1-(4-Aminophenyl)-3-(3-hydroxy-4-methoxyphenyl)prop-2-en-1-one (Compound **4**)^74^

Synthesized from 4-aminoacetophenone and isovanillin. Yellow solid, 53% yield; mp 187–189 °C; IR (neat) cm^–1^: 3465, 3365, 3223, 1629, 1599. ^1^H NMR (500 MHz, DMSO-*d*_6_): δ 3.82 (s, 3H, OC*H*_3_), 6.08 (s, 2H, N*H*_2_), 6.60 (d, *J* = 8.6 Hz, 2H, Ar*H*), 6.97 (d, *J* = 8.3 Hz, 1H, Ar*H*), 7.22 (dd, *J* = 8.3, 1.4 Hz, 1H, Ar*H*, 7.25 (d, *J* = 1.5 Hz, 1H, Ar*H*), 7.48 (d, *J* = 15.4 Hz, 1H, COC*H*=CHAr), 7.60 (d, *J* = 15.4 Hz, 1H, COCH=C*H*Ar), 7.88 (d, *J* = 8.6 Hz, 2H, Ar*H*), 9.09 (br s, 1H, O*H*). ^13^C NMR (125 MHz, DMSO-*d*_6_): δ 55.7, 111.9, 112.7, 114.6, 119.8, 121.4, 125.6, 128.1, 130.9, 141.8, 146.6, 149.7, 153.7, 185.9. HRMS-TOF: [M + Na]^+^ 292.0945 (calcd for C_16_H_15_NNaO_3_: 292.0944).

#### (*E*)-1-(4-Aminophenyl)-3-(3-methoxyphenyl)prop-2-en-1-one (Compound **5**)^48^

Synthesized from 4-aminoacetophenone and 3-methoxybenzaldehyde. Yellow solid, 76% yield; mp 107–108 °C; IR (neat) cm^–1^: 3441, 3341, 3237, 1645, 1579. ^1^H NMR (500 MHz, CDCl_3_): δ 3.85 (s, 3H, OC*H*_3_), 4.17 (br s, 2H, N*H*_2_), 6.70 (d, *J* = 8.2 Hz, 2H, Ar*H*), 6.94 (d, *J* = 7.6 Hz, 1H, Ar*H*), 7.15 (s, 1H, Ar*H*), 7.23 (d, *J* = 7.3 Hz, 1H, Ar*H*), 7.32 (t, *J* = 7.7 Hz, 1H, Ar*H*) 7.52 (d, *J* = 15.6 Hz, 1H, COC*H*=CHAr), 7.74 (d, *J* = 15.6 Hz, 1H, COCH=C*H*Ar), 7.93 (d, *J* = 8.2 Hz, 2H, Ar*H*). ^13^C NMR (125 MHz, CDCl_3_): δ 55.5, 113.5, 114.1, 116.0, 121.0, 122.5, 128.7, 130.0, 131.2, 136.9, 143.2, 151.3, 160.0, 188.2. HRMS-TOF: [M + Na]^+^ 276.1000 (calcd for C_16_H_15_NNaO_2_: 276.0995).

### Preparation of Tested Compounds

Stock solutions were made in the following manner: all five aminochalcones were dissolved in dimethyl sulfoxide (DMSO) (Sigma-Aldrich, Cat. no. 41639) to produce stock solutions. Following that, working solutions were prepared by diluting the stock solution in DMEM containing 10% fetal bovine serum (FBS) and 1% penicillin–streptomycin in appropriate concentrations. Resveratrol (Sigma-Aldrich, Cat. no. R5010–100MG) was used as a reference compound of a recognized SIRT1 activator.^61,70^

### Cytotoxicity Assay

The cytotoxic activity of compounds **1**–**5** was tested using a normal embryonic lung (MRC-5) cell line. MRC-5 cells were grown in DMEM supplemented with 100 U/mL penicillin–streptomycin and 10% FBS. Briefly, the cells suspended in the corresponding culture medium were inoculated in 96-well microtiter plates (Corning Inc., NY, USA) at 10,000–20,000 cells per well and then incubated at 37 °C under a humidified atmosphere with 5% CO_2_ for 24 h. An equal volume of additional medium containing serial dilutions of test compounds, positive control (doxorubicin), or negative control (DMSO) was added to the desired final concentrations. Microtiter plates were further incubated for 48 h. The cell viability in each well was determined by staining with the MTT assay reagent (Molecular Probes, Cat. no. M6494).^75–77^ MTT solution (10 mL/100 mL medium) was added to all wells of the assay, and the plates were incubated for 2–4 h. Subsequently, DMSO was added to dissolve the resulting formazan by sonication. The plates were read on a microplate reader (Molecular Devices, USA) using a test wavelength of 550 nm and a reference wavelength of 650 nm. The IC_50_ value was determined as the concentration of compound that inhibited the cell growth by 50%. The compound showing IC_50_ > 50 μg/mL was considered noncytotoxic.

### Cell Viability Assessment by the MTT Assay

Cell viability was evaluated using the MTT assay. Briefly, 96-well plates were seeded with SH-SY5Y cells at a density of 1 × 10^5^ cells/mL and allowed to attach. Cells were pretreated for 3 h with aminochalcones or resveratrol at various concentrations ranging from 0 to 100 μM before being incubated with 400 μM H_2_O_2_ (Sigma-Aldrich, Cat. no. 31642) for 24 h. Cells treated only with culture media, DMEM with 10% FBS, were used as the control. Each well received 5 mg/mL of MTT solution at the end of the incubation time, and the samples were then incubated at 37 °C in the dark for 2–4 h. Following the removal of the solution, 0.04 N HCl in isopropyl alcohol was added to dissolve the formazan crystals. A microplate reader programed to read at 570 nm was used to detect the MTT formazan product. The absorbance of formazan is directly proportional to the number of viable cells. The viability of the cells was expressed in percentages compared with the viability of the control group.^23^

### Cell Morphology Observation

SH-SY5Y cells were prepared at a concentration of 1 × 10^5^ cells/mL and implanted on a cell culture Petri dish overnight. After adhering, the cells were treated with aminochalcones for 3 h. The culture media was refreshed, followed by an additional 24 h of exposure to 400 μM H_2_O_2_. At the end of incubation, the morphology of treated and untreated cells from various distinct fields was observed and captured using an inverted light microscope (Olympus Corporation, Tokyo, Japan) at ×20 magnification.^78^

### Detection of Intracellular ROS Production

The production of intracellular ROS was revealed using 2′,7′-dichlorofluorescein diacetate (DCFDA) (Molecular Probes, Cat. no. D399), a ROS-sensitive fluorescent probe. Green fluorescent DCF was generated by the oxidation of nonfluorescent carboxy-DCFDA, which is caused by intracellular ROS. SH-SY5Y cells (200 μL) at 1.0 × 10^5^ cells/mL were seeded and incubated overnight in 96-well plates at 37 °C. Then, each well was pretreated for 3 h with 5 μM aminochalcones or resveratrol before exposure to 400 μM H_2_O_2_ for an additional 24 h. Subsequently, carboxy-H_2_DCFDA was introduced to the to a final concentration of 10 μM in the culture media and incubated at 37 °C for 30 min under dark conditions. Intracellular ROS production was instantly evaluated using a microplate reader at an emission wavelength of 528 nm and an excitation wavelength of 485 nm. The intracellular ROS production of the cells was expressed as percentages compared with the control group.^79^

### Measurement of MMP with Rhodamine-123

Mitochondrial-specific fluorescent dye, Rhodamine-123 (Sigma-Aldrich, Cat. no. R8004), was used to determine the MMP. The reduction in the fluorescence signal correlated with the decrease in MMP, which referred to a decrease in the mitochondrial function. SH-SY5Y cells (200 μL) at 1.0 × 10^5^ cells/mL were plated onto the 96-well plate and incubated overnight at 37 °C. Then, each well was pretreated for 3 h with 5 μM aminochalcones or resveratrol before being challenged with 400 μM H_2_O_2_ for further 24 h. Cells were then exposed to Rhodamine-123 at a final concentration of 10 μM in the culture media and incubated at 37 °C for 30 min under dark conditions. At the end of incubation, cells were washed twice with PBS to remove the excess dye. MMP levels were then measured with a microplate reader at excitation and emission wavelengths of 488 and 525 nm, respectively. The MMP of the cells was expressed as percentages compared with the control group.^80^

### Determination of Cell Membrane Damaged by the LDH Assay

LDH leakage into the culture medium of aminochalcones with or without H_2_O_2_-treated cells was assessed by using an LDH assay kit to determine the neuroprotective effect of aminochalcones against H_2_O_2_-induced cell damage. To prepare samples for the LDH test, 2 mL of SH-SY5Y cells at 1.0 × 10^5^ cells/mL concentration was added and incubated overnight in 6-well plates at 37 °C. Then, each well was pretreated for 3 h with 5 μM aminochalcones or resveratrol before exposure to 400 μM H_2_O_2_ for another 24 h. Subsequently, the LDH assay kit (Cat. no. MAK066) from Sigma-Aldrich (St. Louis, MO, USA) was used to measure the LDH activity, following the manufacturer’s instructions. The assay relies on the conversion of lactate to pyruvate by the enzyme lactate dehydrogenase and further produces NADH as a product, giving rise to an increase in absorbance at 450 nm. The LDH activity was represented as a proportion to the control group.^81^

### Measurement of SIRT1 Activity

The SIRT1 assay kit (Cat. no. CS1040–1KT) from Sigma-Aldrich (St. Louis, MO, USA) was used to evaluate the SIRT1 deacetylase activity. SH-SY5Y cells were cultured in 6-well plates overnight, followed by 3 h of pretreatment with 5 μM aminochalcones or resveratrol and another 24 h of exposure to 400 μM H_2_O_2_. Cells were then washed with ice-cold PBS and extracted in a lysis buffer (Cell Signaling Technology, Cat. no. 9806) containing protease inhibitors (Calbiochem, Cat. no. 539131–1VL) at 4 °C for 20 min. Cells were then withdrawn from the growth dish and centrifuged at 12,000 *g* for 20 min at 4 °C. The supernatant was collected, and the amount of protein was determined using the Bradford protein assay (Bio-Rad Laboratories, Cat. no. 5000006) in accordance with the manufacturer’s instructions. The obtained proteins were then analyzed for SIRT1 activity using the SIRT1 assay kit performed according to the manufacturer’s protocol. SIRT1 activity was measured with a microplate reader at excitation and emission wavelengths of 340 and 445 nm, respectively. The SIRT1 activity of cells was expressed as percentages relative to the control.^81^

### *In Silico* Prediction of Pharmacokinetic Properties

To anticipate drug-likeness and potential toxicity of the studied aminochalcones, pharmacokinetic properties (i.e., ADME) and toxic features of compounds **1**–**5** were predicted using web-based tools including SwissADME (http://www.swissadme.ch/),^53^ pkCSM (http://biosig.unimelb.edu.au/pkcsm/),^82^ and ProTox-II (http://tox-new.charite.de/protox_II/).^83^ Chemical structures of the compounds in SMILES format were uploaded to the web server as an input data set for prediction. The compounds were assessed drug-likeness-based Veber’s rule, and Lipinski’s rule of five (LRo5).

### Molecular Docking

Molecular docking was performed to identify potential binding modalities of four studied aminochalcones (**1**–**3** and **5**) with the NAD-dependent deacetylase sirtuin-1 (SIRT1)^64,66^ using AutoDockTools v.4.2.6.^84^ The crystallographic structure at 3.2 Å resolution of a human SIRT1 in its activated state, which forms a complex with cocrystallized ligands (specifically, three molecules of resveratrol), and an AMC-containing peptide was retrieved from the PDB (5BTR).^52^ The cocrystallized ligands were removed from the target protein before being docked, and only chain A was chosen. The polar hydrogen atoms and charges were then added to the protein using the AutoDockTools. A grid spacing of 0.375 Å and a grid box size (50 × 50 × 50 points) were applied as the docking parameters. To guarantee coverage of the whole activator-binding interface between the SIRT1 C- and N-terminal domains, the grid box center was positioned at −23.315, 65.890, and 14.723. Rotational bonds in the protein structures were classified as rigid, whereas rotational bonds in the compound structures were assumed as flexible. The number of independent docking runs for each docking simulation was set at 100 runs, and the Lamarckian Genetic Algorithm was used as the search parameter, with the maximum amount of energy adjusted to the medium level.^85^ The structural difference between cocrystallized and redocked resveratrol molecules was determined by computing the root-mean-square deviation to validate the reliability of docking process. Following the docking process, the binding behavior and key binding interactions between the examined compounds and the target SIRT1 were revealed and illustrated using the Discovery Studio Visualizer 2016 (BIOVIA, Dassault Systèmes, CA, USA).^81^

### Quantification and Statistical Analysis

GraphPad Prism 6 (GraphPad Software Inc., CA, USA) was employed to calculate statistical comparisons between groups using one-way analysis of variance (ANOVA), followed by a Tukey–Kramer post-hoc test. The outcomes are presented as mean ± SEM of three or more independent experiments, and the probability (*P*) value of <0.05 is regarded statistically significant.

## Supporting Information Available

Scheme of *para*-aminochalcones synthesis by the Claisen–Schmidt condensation of 4-aminoacetophenone **A** and the corresponding benzaldehydes **B** with NaOH (PDF)

## Abbreviations

BBB: blood brain barrier
CNS: central nervous system
DCFDA: 2′-7′-dichlorodihydrofluorescein diacetate
DMEM: Dulbecco’s Modified Eagle's Medium
H_2_O_2_: hydrogen peroxide
LDH: lactate dehydrogenase
MTT: 3-(4,5-dimethylthiazol-2-yl)-2,5-diphenyltetrazolium bromide
MMP: mitochondrial membrane potential
SIRT1: Sirtuin 1, also known as NAD-dependent deacetylase sirtuin-1
