# Supplementary material for: Aminochalcones Attenuate Neuronal Cell Death under Oxidative Damage via Sirtuin 1 Activity
Source: ACS Omega. 2023 Sep 7;8(37):33367–79. doi: 10.1021/acsomega.3c03047 (PMC10515382; doi:10.1021/acsomega.3c03047)
Supplement: Supplementary file 1 — ao3c03047_si_001.pdf [file ao3c03047_si_001.pdf]

Supporting Information for Publication: Supporting information

Aminochalcones Attenuate Neuronal Cell Death under Oxidative Damage via the Sirtuin 1 Activity

Setthawut Apiraksattayakul[1], Ratchanok Pingaew[2*], Ronnakorn Leechaisit[2], Veda Prachayasittikul[1], Waralee Ruankham[1], Napat Songtawee[3], Tanawut Tantimongcolwat[1], Somsak Ruchirawat[4,5], Virapong Prachayasittikul[6], Supaluk Prachayasittikul[1], and Kamonrat Phopin[1,6*]

[1]Center for Research Innovation and Biomedical Informatics, Faculty of Medical Technology, Mahidol University, Bangkok 10700, Thailand

[2]Department of Chemistry, Faculty of Science, Srinakharinwirot University, Bangkok 10110, Thailand

[3]Department of Clinical Chemistry, Faculty of Medical Technology, Mahidol University, Bangkok 10700, Thailand

[4]Laboratory of Medicinal Chemistry, Chulabhorn Research Institute, and Program in Chemical Science, Chulabhorn Graduate Institute, Bangkok 10210, Thailand

[5]Center of Excellence on Environmental Health and Toxicology (EHT), Commission on Higher Education, Ministry of Education, Bangkok 10400, Thailand

[6]Department of Clinical Microbiology and Applied Technology, Faculty of Medical Technology, Mahidol University, Bangkok 10700, Thailand

*Email: ratchanok@g.swu.ac.th Phone: +66 (2) 649 5000, Fax: +66 (2) 260 0128

*Email: kamonrat.php@mahidol.ac.th, kamonrat.php@mahidol.edu.

Phone: +66 (2) 441 4371, Fax: +66 (2) 441 4380

Scheme of *para*-aminochalcones synthetization by the Claisen–Schmidt condensation of 4-aminoacetophenone **A** and the corresponding benzaldehydes **B** with NaOH. [1-3]

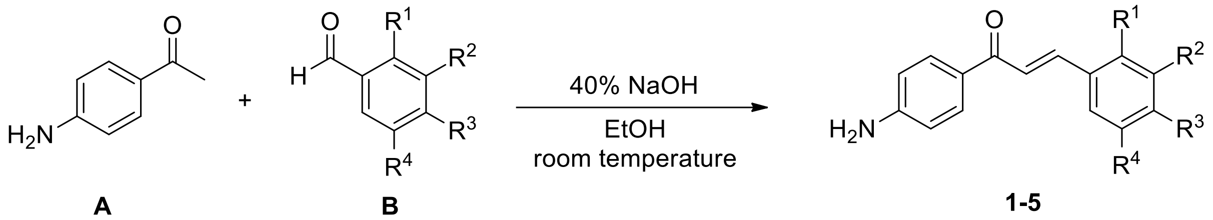

Scheme S1. Synthesis of aminochalcones **1–5**.

REFERENCE

(1) Sooknual, P.; Pingaew, R.; Phopin, K.; Ruankham, W.; Prachayasittikul, S.; Ruchirawat, S.; Prachayasittikul, V. Synthesis and neuroprotective effects of novel chalcone-triazole hybrids. *Bioorg. Chem.* **2020**, *105*, 104384. DOI: https://doi.org/10.1016/j.bioorg.2020.104384.

(2) Pingaew, R.; Saekee, A.; Mandi, P.; Nantasenamat, C.; Prachayasittikul, S.; Ruchirawat, S.; Prachayasittikul, V. Synthesis, biological evaluation and molecular docking of novel chalcone–coumarin hybrids as anticancer and antimalarial agents. *Eur. J. Med. Chem.* **2014**, *85*, 65-76. DOI: https://doi.org/10.1016/j.ejmech.2014.07.087.

(3) Ganji, L. R.; Gandhi, L.; Musturi, V.; Kanyalkar, M. A. Design, synthesis, and evaluation of different scaffold derivatives against NS2B-NS3 protease of dengue virus. *Med. Chem. Res.* **2021**, *30* (1), 285-301. DOI: https://doi.org/10.1007/s00044-020-02660-y.
